# Characterisation of the Upper Respiratory Tract Virome of Feedlot Cattle and Its Association with Bovine Respiratory Disease

**DOI:** 10.3390/v15020455

**Published:** 2023-02-06

**Authors:** Rebecca K. Ambrose, Claudia Blakebrough-Hall, Jennifer L. Gravel, Luciano A. Gonzalez, Timothy J. Mahony

**Affiliations:** 1 Agriscience Queensland, Department of Agriculture and Fisheries, Ecosciences Precinct, Dutton Park, Brisbane, QLD 4102, Australia; 2 School of Life and Environmental Sciences, Faculty of Science, University of Sydney, Sydney, NSW 2570, Australia; 3 Sydney Institute of Agriculture, University of Sydney, Biomedical Building, Australian Technology Park, Sydney, NSW 2015, Australia; 4 Queensland Alliance for Agriculture and Food Innovation, Queensland Bioscieces Precinct, The University of Queensland, Brisbane, QLD 4072, Australia

**Keywords:** bovine respiratory disease, virome, bovine nidovirus, bovine coronavirus, bovine herpesvirus 1, bovine viral diarrhoea virus 1, bovine respiratory syncytial virus, case control, odds ratio

## Abstract

Bovine respiratory disease (BRD) is a major health problem within the global cattle industry. This disease has a complex aetiology, with viruses playing an integral role. In this study, metagenomics was used to sequence viral nucleic acids in the nasal swabs of BRD-affected cattle. The viruses detected included those that are well known for their association with BRD in Australia (bovine viral diarrhoea virus 1), as well as viruses known to be present but not fully characterised (bovine coronavirus) and viruses that have not been reported in BRD-affected cattle in Australia (bovine rhinitis, bovine influenza D, and bovine nidovirus). The nasal swabs from a case–control study were subsequently tested for 10 viruses, and the presence of at least one virus was found to be significantly associated with BRD. Some of the more recently detected viruses had inconsistent associations with BRD. Full genome sequences for bovine coronavirus, a virus increasingly associated with BRD, and bovine nidovirus were completed. Both viruses belong to the *Coronaviridae* family, which are frequently associated with disease in mammals. This study has provided greater insights into the viral pathogens associated with BRD and highlighted the need for further studies to more precisely elucidate the roles viruses play in BRD.

## 1. Introduction

Bovine respiratory disease (BRD) is the most significant health problem within the feedlot industry. Despite advances in veterinary medicine and improvements in control measures, BRD remains a major economic burden for the beef industry through reduced growth rates, mortality, and organ condemnation, while increasing treatment and labour costs [1]. The pathogenesis of BRD is complex, with several viruses, bacteria, host, and environmental factors contributing to its onset [2,3,4,5,6,7,8,9,10,11]. The viruses historically associated with BRD are bovine alphaherpesvirus 1 (BoHV-1), bovine viral diarrhoea virus 1 (BVDV-1), bovine parainfluenza virus 3 (BPI-3), and bovine respiratory syncytial virus (BRSV) (Fulton, 2020). Bacterial infections (predominantly *Mannheimia haemolytica*, *Pasteurella multocida*, *Histophilus somni*, and *Mycoplasma bovis*) are generally considered to be secondary pathogens, opportunistically colonising the respiratory mucosa following the damage caused by the primary viral infection or because of immunosuppression [9,12,13].

Viral metagenomics, using next-generation sequencing (NGS) technologies, has recently allowed for the rapid genetic characterisation of viral genetic material in clinical samples, the virome, and has revealed the presence of both known and novel viruses in healthy and sick animals and people [14,15,16,17]. Unlike conventional diagnostics, this technology does not require prior knowledge of the genetic information of a pathogen for it to be detected, hence allowing for the unbiased assessment of clinical samples and the discovery of novel viruses. Although the pathogens listed previously are thought to be the principal pathogens associated with BRD, recent viral metagenomics studies suggest that the repertoire of viruses associated with BRD is more diverse, which may be a contributing factor to the failure to manage this disease adequately [14,18].

The first aim of the current study was to characterise the virome present in the nasal swabs of feedlot cattle treated for BRD. The second aim of the study was to use virus specific-qPCR assays informed by the virome to determine the presence and absence of viral genetic material in the nasal swabs taken from BRD-affected and -unaffected cattle to determine the associations between these viruses and the risk of animals developing the disease.

## 2. Materials and Methods

### 2.1. Viral Metagenomics

Nasal swabs used in this study were collected as part of the National Bovine Respiratory Disease Initiative (NBRDI), which was a nationwide prospective longitudinal study conducted in Australia to evaluate possible risk factors for BRD in feedlot cattle [7]. Briefly, nasal swabs (15.2 cm, without transport media) were collected from cattle treated for BRD with signs of respiratory disease. On receipt at the laboratory, the swabs were added to a 96 well-plate containing 500 µL phosphate-buffered saline (PBS) containing 5× antibiotic/antimycotic (ThermoFisher Scientific, Waltham, MA, USA). Samples were stored at −80 °C until required.

Six pools consisting of six randomly selected nasal swab samples were prepared using 50 µL from each nasal swab sample from the NBRDI study population [6]. The 300 µL pooled samples were passed through a 200 nm filter (Merck, Kenilworth, NJ, USA) to remove eukaryotic cells, bacteria, and particulate debris. The resulting filtrate was incubated at 37 °C for 90 min in a cocktail of 14 U Turbo DNase (Ambion, Berlin, Germany), 25 U Benzonase^®^ (Sigma Aldrich, St. Louis, MO, USA) and 20 U RNase1 (ThermoFisher Scientific, Waltham, MA, USA) to degrade the host (bovine) or unprotected environmental nucleic acids. Viral RNA was isolated using the QIAamp MinElute Virus Spin kit^®^ (Qiagen, Venlo, The Netherlands) according to the manufacturer’s instructions.

Complementary DNA (cDNA) of the RNA in the extract was prepared by reverse transcription using an oligonucleotide, containing a specific nucleotide sequence (5′ residues 1 to 20) and a random sequence with 8Ns (residues 21 to 28) at the 3′ end (cDNA primer: 5′-CCTTGAAGGCGGACTGTGAGNNNNNNNN-3′) [14] using the Superscript III reverse transcription kit (ThermoFisher Scientific, Waltham, MA, USA) according to the manufacturer’s instructions. Second-strand synthesis was performed using Klenow fragment DNA polymerase (New England Biolabs, Ipswich, MA, USA) and the cDNA primer so that the complementary strand of the cDNA also encoded the fixed portion of the cDNA primer at the 5′ terminus. The resulting double-stranded cDNA was PCR amplified using Platinum™ Taq DNA polymerase (ThermoFisher Scientific, Waltham, MA, USA) and the oligonucleotide amplification primer: 5′-CCTTGAAGGCGGACTGTGAG-3′ [15]. The 50 µL reaction mix contained 0.2 mM of each dNTP, 1.5 mM MgCl_2_, 0.2 µM of each primer, and 1.0 U of polymerase. Amplification conditions were as follows: 95 °C for 5 min; 5 cycles of 95 °C for 1 min, 59 °C for 1 min, 72 °C for 1 min; 33 cycles of 95 °C for 20 s, 59 °C for 20 s, and 72 °C for 1 min, increasing by 2 s per cycle; final extension of 72 °C for 7 min.

Amplicons were purified using Qiagen MinElute PCR Purification kit (Qiagen^®^, Venlo, The Netherlands) and then submitted to the Australian Genome Research Facility (AGRF, Melbourne, Australia) for library preparation and NGS. The nucleic acid was subjected to Nextera XT library preparation protocol (Illumina, San Diego, CA, USA) and sequenced using Illumina’s MiSeq platform to generate 300 nucleotide (nt) paired-end reads.

Sequence data were initially quality filtered to remove the low-quality sequences, reads of less than 36 bp, and the Illumina-specific sequencing adaptors using the Trimmomatic program [19]. Trimmed reads were mapped to the host reference genome (*Bos taurus*: bosTau7) and the Illumina quality control template (PhiX174), and unmapped reads were retained for further analysis. Mapping was performed using Bowtie2 [20] with default parameter settings. De novo assembly was completed using Velvet Optimiser and performed using unmapped reads to generate contiguous sequences (contigs) [21,22]. Sequence identity searches were performed with these resulting assembled contigs using BLASTN [23,24] against selected databases. A custom database was constructed based on possible BRD-associated viral sequences determined through the literature. The National Center for Biotechnology Information (NCBI) (https://www.ncbi.nlm.nih.gov, accessed on 19 December 2022) viral reference sequence database and The Nucleotide (nr/nt) database were also used.

Following BLAST analysis, the contigs that were identified as viruses were further analysed using the alignment and mapping programs within MEGA7 [25] and Geneious 9 (http://www.geneious.com, accessed on 19 December 2022) [26]. Contigs were mapped to viral reference genomes to generate consensus sequences and to assess the genome coverage of individual viruses. Phylogenetic and molecular evolutionary analyses were conducted using MEGA version 7 [27].

### 2.2. PCR and Sequencing

For bovine coronavirus (BCoV) and bovine nidovirus (BNV), near-complete genome sequences were obtained following the mapping of NGS reads to the reference genomes. To generate sequence data to fill in the remaining gaps, oligonucleotides were designed, encompassing regions for which no or poor sequence was obtained. The PrimerQuest software (Integrated DNA Technologies, Inc., Coralville, IA, USA) and the newly determined genome sequences were used to design these oligonucleotides. For BCoV, eight oligonucleotide pairs were designed with amplicons varying in size from 193 nt to 2596 nt. For BNV, oligonucleotides (12 pairs) were designed to facilitate amplification across the entire genome with amplicon sizes ranging from 1032 nt to 2518 nt (Supplemental Table S1).

Sample pools (cDNA), for which BCoV and BNV NGS sequence data were obtained, were used as a template for PCR. BCoV PCR was performed using Platinum^®^ Taq Hot-Start DNA polymerase (ThermoFisher Scientific, Waltham, MA, USA). The 25 µL reaction mix contained 0.2 mM of each dNTP, 1.5 mM MgCl_2_, 0.8 µM of each primer, and 1.0 U Taq DNA polymerase. Amplification parameters: initial denaturation at 94 °C for 2 min followed by 40 cycles of denaturation at 94 °C for 30 s, annealing at 55 °C for 30 s, and extension at 72 °C for 1 min per kb. The reaction concluded with a 5 min extension time at 72 °C.

For BNV, Phusion Green Hot-Start II High-Fidelity PCR Master Mix (ThermoFisher Scientific, Waltham, MA, USA) was used according to the manufacturer’s instructions, with a 25 µL total volume. Amplification parameters were initial denaturation at 98 °C for 30 s followed by 40 cycles of denaturation at 98 °C for 10 s, annealing at 55 °C for 30 s, and extension at 72 °C for 1 to 2 min, depending on the size of the expected product. The reaction concluded with a 10 min extension time at 72 °C.

Amplified products were run on a 1% agarose gel stained with Midori green and visualised with a UV transilluminator. Amplicons consistent with the expected sizes were excised from the gel and purified using the QIAquick^®^ Gel Extraction Kit (Qiagen, Venlo, The Netherlands). Direct sequencing of each amplicon was performed using BigDye^®^ Terminator v3.1 (Applied Biosystems™, Foster City, CA, USA), according to the manufacturer’s instructions, and submitted to a commercial sequencing service for fragment analyses (Genetics Research Services, The University of Queensland, St Lucia, Australia).

### 2.3. Quantitative Real-Time PCR (qPCR)

Five sets of amplification oligonucleotides and corresponding dual-labelled hydrolysis probes were designed to detect four RNA viruses and one DNA virus that had not been reported in Australian cattle treated for BRD previously. Published studies have suggested the selected viruses may play a role in the development of BRD in feedlot cattle: BNV, bovine rhinitis A virus (BRAV), bovine rhinitis B virus (BRBV), influenza D virus (IDV), and ungulate bocaparvovirus 6 (UBPV6) [18,28,29,30]. Briefly, in addition to using the sequence data generated with NGS, available nucleotide sequences for these viruses were retrieved from GenBank (https://www.ncbi.nlm.nih.gov/genbank/, accessed on 1 September 2017) and were aligned using MEGA7 [25] to identify the conserved regions suitable for oligonucleotide and hydrolysis probe design. The PrimerQuest software (Integrated DNA Technologies, Inc., Coralville, IA, USA) was used to design the oligonucleotide pairs and corresponding dual-labelled hydrolysis probes and their specificities were evaluated using the BLAST algorithm [31]. The dual-labelled probes had unique reporter dyes/fluorophores at their 5′ ends and Black Hole Quenchers^®^ or Iowa Black^®^FQ at their 3′ ends. For BRBV, for which minimal sequence data was available across conserved regions, the primer-probe set from a published qPCR assay was used [32]. The nucleotide sequences, fluorophores, and quenchers of the oligonucleotide pairs and dual-labelled hydrolysis probes are shown in Table 1.

The specific assays were evaluated using the viral RNA pools and the individual viral RNA samples used for the pools. The exception was UBPV-6, for which the assay was optimised using synthetic double-stranded DNA fragment (gBlocks™ Gene Fragment—Integrated DNA Technologies, Inc., Coralville, IA, USA), as there was insufficient quantity of this virus in the RNA pools.

The assays were further tested using 60 nasal swab extracts from cattle with BRD from feedlots (same feedlots that were used in pools). Viral RNA was extracted from these swabs using the QIAamp-MinElute Virus Spin kit (Qiagen, Venlo, The Netherlands), and qPCR was performed as described below.

As part of the assay evaluation, each primer-probe set was also tested in a reaction with the templates that contained viruses other than the ones they were targeting.

### 2.4. Case Control Study

The case–control analysis was part of a larger study to predict BRD outcomes in feedlot cattle using latent class analysis, which has previously been described [33]. Briefly, the study was conducted at a commercial feedlot in southern New South Wales, Australia, with cattle (*Bos taurus* castrated males, approximately 12–24 months old) sourced from saleyards or cattle backgrounding properties. Following induction, the animals were checked daily by trained feedlot staff for visual signs of BRD. Animals were scored for visual signs of BRD in the pen using a modified version of the Wisconsin calf-scoring chart, which included the assessment of seven visual signs: lethargy, head carriage, laboured breathing, cough, nasal discharge, ocular discharge, and rumen fill [34]. Each clinical sign was assigned a score from 0 to 3, with 3 being the most severe. A case was defined as an animal with a score >0 for at least one of the visual signs specific to BRD: nasal or ocular discharge, laboured breathing, or coughing. For each animal identified with BRD (case), an animal (control) exhibiting no visual signs of BRD (a score of 0 for all the seven visual signs) was removed from the same pen on the same day. Detailed information on the animals used, their management, BRD monitoring, and clinical data collection was previously described [34].

A total of 288 nasal swabs from the study cattle were collected for analysis (141 cases and 147 controls). On receipt in the laboratory, nasal swabs were resuspended in 500 µL PBS. Total nucleic acid was extracted from 200 µL nasal swab sample using the DNeasy 96 Blood and Tissue kit (Qiagen^®^, Venlo, The Netherlands) according to the manufacturer’s instructions. The optional addition of RNaseA was omitted to permit the copurification of RNA and DNA.

Quantitative real-time PCR (qPCR) was performed on these samples to test for the presence of genetic material for BoHV-1, BVDV-1, BRSV, BPI-3, BCoV, BNV, BRAV, BRBV, IDV, and UBPV-6. The qPCR assay for the detection of BoHV-1, BCoV, BRSV, and BPI-3 was performed as a multiplex reaction, as described previously, with BCoV replacing BVDV-1 [35]. Detection of BRAV, BRBV, and IDV was also performed as a multiplex reaction with primer and probe concentrations of 0.4 µM and 0.2 µM, respectively. BVDV-1 and BNV were detected using singleplex assays, with primer and probe concentrations of 0.6 µM and 0.2 µM, respectively. All viral RNA assays were performed using the QuantiTect Multiplex RT-PCR Kit (Qiagen, Venlo, The Netherlands) and Qiagen Rotor-Gene^®^ Q machine. The reactions were performed as per the manufacturer’s instructions. For UBPV-6, qPCR was performed using IDT PrimeTime^®^ Gene Expression Master Mix according to manufacturer’s protocol and with primer and probe concentrations as per the singleplex assays above. Samples were considered positive if the threshold cycle (Ct) value was ≤35.

Odds ratios (OR) were calculated to measure the association between virus detection and clinical signs of BRD. OR confidence intervals (CI) were used to estimate the precision of the OR, and *p* values were also calculated from the CI. Statistical significance was defined as *p* < 0.05. The OR, its standard error, 95%CI and *p* value were calculated as described by [36,37].

To test the hypothesis that the Ct values for each virus detected in the nasal swabs collected from animals with clinical signs of BRD was the same as the Ct values from samples collected from the asymptomatic animals, a *t*-test was performed to compare the means of the two groups. Comparisons yielding *p* < 0.05 were considered statistically significant. This statistical method was used to test the hypothesis that the Ct values for IDV in those animals coinfected with another virus is the same as the Ct values in those animals infected with IDV alone.

## 3. Results

### 3.1. Viral Metagenomics

The nasal swabs collected from the 36 animals treated for BRD were pooled into six pools of six animals and were deep sequenced using the Illumina MiSeq platform. A total of 17,195,238 pair-ended 300 nt sequence reads (average 2,865,873 per sample) were generated. Following quality control, de novo assembly was performed. BLAST searches of the resulting contigs against a custom BRD viral database, a viral reference sequence database (NCBI), and a nonredundant sequence database (NCBI) identified several contigs in the experimental datasets with high identity to viruses in four of the six sample pools. The two pooled samples where no viral sequences were identified were not analysed further.

In the data from the remaining samples, sequences from viruses from the following families were identified: *Coronaviridae*; *Tobaniviridae*; *Flaviviridae*; *Orthomyxoviridae; Picornaviridae*; and *Parvoviridae*. Contigs were mapped to reference viral genome sequences using Geneious^®^ (Version 9), which resulted in the generation of near-complete and partial viral genome sequences for a subset of the viruses. Further analyses were undertaken, including phylogenetic analyses, to evaluate the relationships of these newly sequenced viruses to those viruses present in the databases.

#### 3.1.1. Orthomyxoviridae

Viral sequences with high identity to IDV were identified in the data from one of the four pools. The sequence comparisons demonstrated that four of the seven genomic segments were represented in the dataset. Each of the four segments was assembled using the respective sequences from the IDV strain D/bovine/Miyazaki/B22/2016 as guide templates. The lengths of each segment identified in the sequencing data and the coverage of the respective fragments compared to the reference strain are summarised in Table 2. Segment 1 was the most complete, with a length of 1054 nt, covering 44.6% of the analogous segment from the reference strain (Table 2). Overall, there were very high sequence identities and similarities at the nucleotide and amino acid levels, respectively (Table 2). These values were reduced for segment 4 (encoding haemagglutinin-esterase), although it should be noted that it also had the lowest coverage of 12.1% (Table 2).

**Figure 1:**
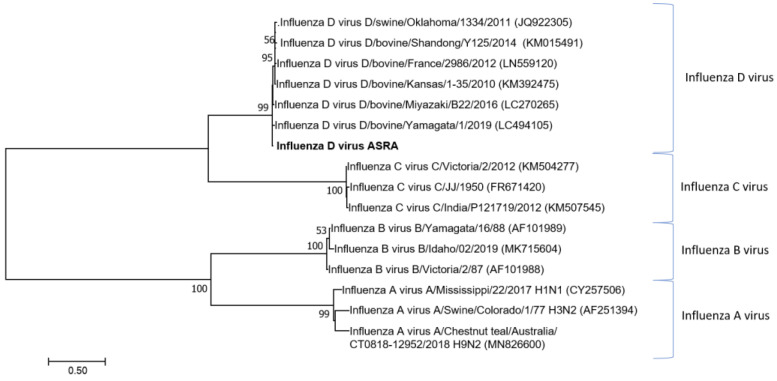
Phylogenetic tree of the influenza D virus based on the polymerase PB2 gene. The phylogenetic tree was constructed using the maximum likelihood method based on the Tamura-Nei model [38,39]. Bootstrapping of 1000 replicates was performed. To determine the best model to use for the phylogenetic analysis, model selection was performed, which analysed the maximum likelihood fits of 24 different nucleotide substitution models. The trees are drawn to scale with the scale, bar representing the number of nucleotide substitutions per site. The numbers at the nodes represent percentage bootstrap support (values are indicated for each node >50%). The Australian sequences from this study are shown in bold. The isolate names and GenBank accession numbers for sequences used in the trees are shown.

Maximum likelihood phylogenetic analysis of the partial nucleotide sequences of the IDV segment 1 (Figure 1) demonstrated that this newly identified viral sequence clusters with other IDV strains and is distinct from other genera in the *Orthomyxoviridae* family. The inferred relationship to other IDV strains and tree topology were supported by high bootstrap scores.

#### 3.1.2. Coronaviridae

**Figure 2:**
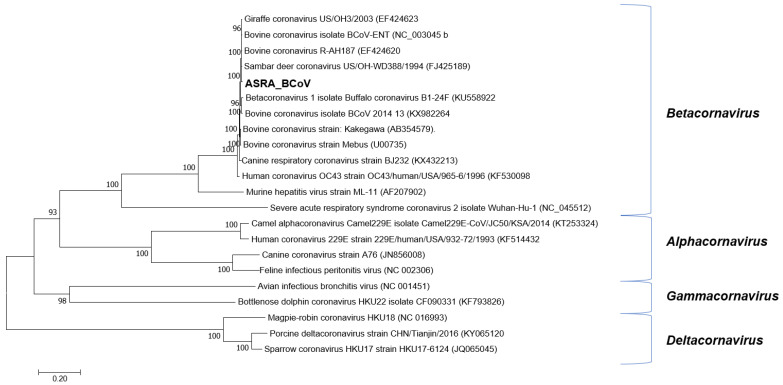
Phylogenetic tree of *Coronaviridae* based on the ORF1ab gene. The phylogenetic tree was constructed using the maximum likelihood method based on the general time reversible model. Bootstrapping of 500 replicates was performed. Phylogenetic analysis of the predicted nucleotide sequences determined in this study. In order to determine the best DNA model to use for phylogenetic analysis, model selection was performed, which analysed the maximum likelihood fits of 24 different nucleotide substitution models. The trees are drawn to scale, with the scale bar representing the number of nucleotide substitutions per site. Numbers at nodes represent percentage bootstrap support (values are indicated for each node >50%). The Australian sequences from this study are shown in bold. The isolate names and GenBank accession numbers for the sequences used in the trees are shown.

Following de novo assembly, 255 contigs were identified in one sample pool, having an identity to the subfamily *Coronavirinae*. These contigs were mapped to the genome of BCoV-ENT (GenBank accession number NC_003045, [40], resulting in near-complete genome coverage. These regions were amplified by RT-PCR using the same sample pool extract used for the NGS library construction and were directly sequenced using conventional dideoxy-terminator technology to complete the genome sequence. The first completed BCoV genome sequence from Australia (BCoV-Aus) was 30,999 nt in length, with a G + C content of 36.9%. The BCoV-Aus genome sequence demonstrated greater than 98% nucleotide identity to other BCoV genomes in GenBank. The genome organisation was typical of the BCoVs and nucleotide identities between the BoCV reference genome sequence strain BCoV-ENT and buffalo coronavirus (accession number: KU558923 [41]), to which it had the highest nucleotide sequence identity, as shown in Table 3. Phylogenetic reconstructions based on the ORF1ab gene demonstrated robust clustering with these viruses (Figure 2). The BCoV genome deduced in this study encoded five putative accessory (nonstructural) proteins characteristic of BCoVs [42]. In comparison with existing BCoV sequences, the analysis of the BCoV sequence deduced in this study identified that the open reading frames (ORFs) encoding the 4.8 kDa and 4.9 kDa nonstructural proteins were truncated, resulting in smaller proteins than expected [40,43]. The 4.9 kDa protein encoded by the BCoV characterised in the current study was 25 amino acids (aa) in length rather than the expected 29 aa [40]. Similarly, the 4.8 kDa protein was predicted to be reduced to 29 aa in length compared to the expected 45 aa. Similar changes to both these proteins were also evident in two buffalo coronaviruses (B1-28F and B1-24F), with which the BCoV in this study shows high identity. Both coronaviruses had shorter 4.9 kDa proteins (25aa), and the 4.8 kDa protein was 29 aa in length for B1-24F and 44 aa for B1-28F.

#### 3.1.3. Tobaniviridae

Following de novo assembly, 612 contigs were identified with identity to BNV. At the time of this analysis, there was one full genome sequence available in GenBank for comparison: BNV strain TCH5 (GenBank Accession NC_027199) [28]. Oligonucleotide pairs were designed to amplify the PCR amplicons spanning putative gaps in the viral genome, which were identified after mapping the contigs to the reference sequence. The addition of these amplicon sequences to the genome assembly and mapping to the reference genome sequence resulted in a full-length genome sequence that was 20,262 nt in length. The NGS-derived genome sequence has 85.9% identity to the reference BNV genome [28].

Genome annotation revealed genome organisation that was consistent with the BNV previously reported, with a large replicase polyprotein and several shorter downstream ORFs. Nucleotide identities for the genes ranged from 75.6% for the glycoprotein G2 to 94.8% for the hypothetical protein (Table 4). Further sequencing and analysis of more Australian BNV isolates is required to elucidate the genome sequence more accurately.

**Figure 3:**
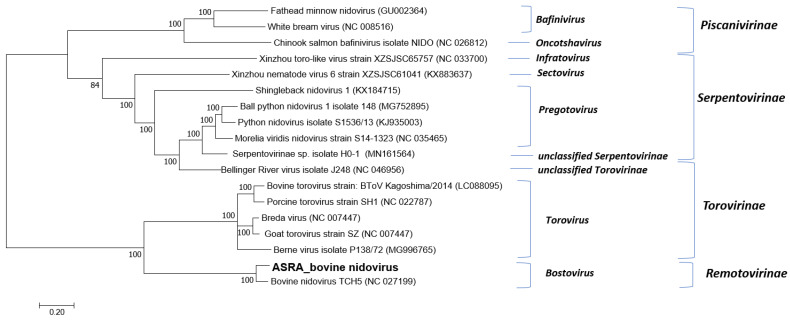
Phylogenetic tree of the family *Tobaniviridae* based on the replicase polyprotein (pp1a/b) gene. The phylogenetic tree was constructed using the maximum likelihood method based on the general time reversible model. Bootstrapping of 500 replicates was performed. Phylogenetic analysis of the predicted nucleotide sequences determined in this study. In order to determine the best DNA model to use for phylogenetic analysis, model selection was performed, which analysed the maximum likelihood fits of 24 different nucleotide substitution models. The trees are drawn to scale, with the scale bar representing the number of nucleotide substitutions per site. Numbers at nodes represent percentage bootstrap support (values are indicated for each node >50%). The Australian sequences from this study are shown in bold. The isolate names and GenBank accession numbers for the sequences used in the trees are shown.

Multiple sequence alignment and phylogenetic analysis clearly demonstrated that, for this virus group with the BNV TCH5 is a member of the *Bostovirus* genus within the subfamily *Remotovirinae* (Figure 3).

#### 3.1.4. Flaviviridae

**Figure 4:**
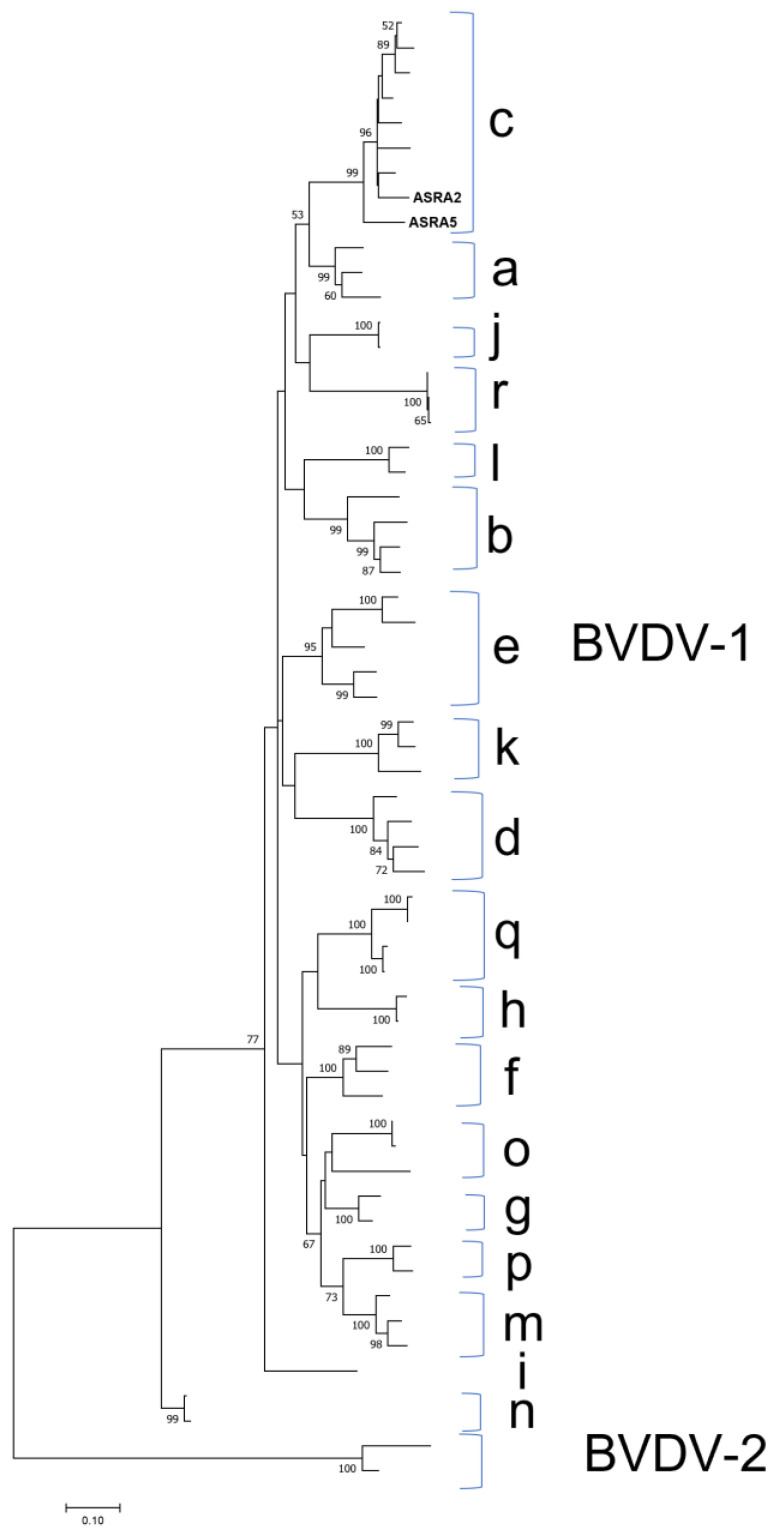
Phylogenetic tree of Bovine Viral Diarrhoea Virus 1 (BVDV-1) based on the N^pro^ gene for the sequences from this study (ASRA2 and ASRA5). The phylogenetic tree was constructed using the maximum likelihood method based on the Kimura 2-parameter model [27]. Parentheses indicate the clustering of representative BVDV-1 isolate sequences into recognised genotype a to genotype n. The phylogenetic tree is rooted to N^pro^ gene sequences of BVDV-2. The sequence accession numbers used in the reconstruction for each genotypes were: BVDV-1c AY763093, KF896608, AY763095, AY763094, AY182162, AF144464, AY182160; BVDV-1a EU180034, M96751, M31182; j U80902, AB078950; BVDV-1r AB078950, KF154779, KF154777; BVDV-1l EU163964, EU163950; BVDV-1b U63479, AY182155, AF287280, M96687; BVDV-1e AF287281, AY735490, KP313732, AF287282, AY735489; BVDV-1k AY894998, AY894997, EU180037; BVDV-1d AF144473, AF144462, AF287284, AF144463; BVDV-1q JN400273, KC695812, KC695811, KC695810; BVDV-1h AF287285, EU163971; BVDV-1f AF287290, AF287286, EU163974; BVDV-1o AB359932, AB359931, KC207073; BVDV-1g AF287287, AF287283; BVDV-1p GU120259, KC207071; BVDV-1m KR866116, AF526381, KC207075; BVDV-1i AF287279; BVDV-1n AB359929, AB359930. BVDV-2: AF104030, U18059. Bootstrapping of 1000 replicates was performed.

There were 509 BVDV-1 contigs identified in two of the analysed samples. Two consensus sequences were generated following the mapping of these contigs to the Australian Bega isolate (accession number: KF896608). The consensus sequences covered 96.4% and 96.3% of the Bega isolate genome (a non-cytopathogenic strain) with a sequence identities of 90.0% and 90.9%, respectively. The phylogenetic analysis demonstrated that this genome sequence clusters with other BVDV-1c isolates (Figure 4). The 1c genotype is the most reported genotype in the Australian cattle population [44,45].

#### 3.1.5. Picornaviridae

**Figure 5:**
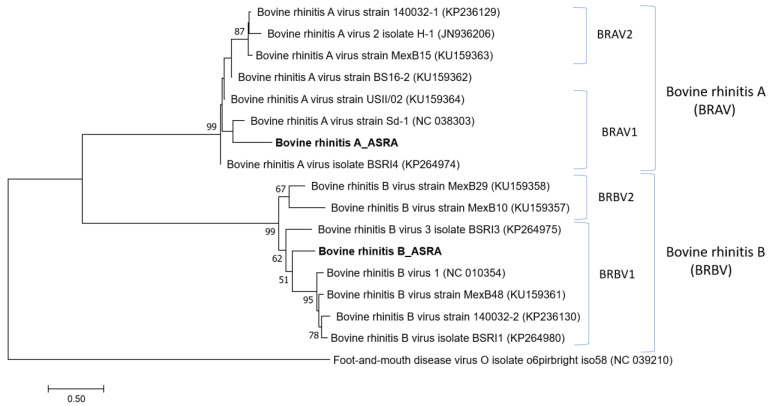
Phylogenetic tree of *Aphthovirus* based on the polyprotein gene. The phylogenetic tree was constructed using the maximum likelihood method based on the Kimura 2-parameter model [27]. Bootstrapping of 1000 replicates was performed.

Several contigs (22) with sequence identity to bovine rhinitis A virus (BRAV) and bovine rhinitis B virus (BRBV) were detected. The alignment of these contigs to representatives of the BRAV and BRBV genomes (~7500 nt in length) produced consensus sequences covering 50.8% and 23.7% of the BRAV and BRBV genomes, respectively. Although there was only 80% nucleotide identity with the reference BRAV and BRBV genomes, the phylogenetic reconstruction strongly supported that the viral sequences identified in this study cluster with their respective viruses in the *Apthovirus* genus (Figure 5). The genetic clustering and overall phylogenetic tree topology were supported by high bootstrap scores. Further work is required to obtain more comprehensive sequence data for these viruses in the Australian cattle population.

#### 3.1.6. Parvoviridae

Although sample preparation was optimised for the preferential sequencing of RNA viruses, several viruses with small DNA genomes belonging to the *Parvoviridae* family were also detected. This may be due to a small amount of viral DNA remaining post-DNase treatment, which was subsequently amplified prior to library preparation. Alternatively, the sample extracts may have contained transcripts from these viruses. Several sequences corresponding to three viral genera within this family were identified.

- *Bocoparvovirus*

Six contigs (206 to 351 bases in length) were identified that were most closely related to viruses in the *Bocaparvovirus* genus. One contig was 100% identical over a 205 nt region of the ORF2 structural protein of the reference ungulate bocaparvovirus 6 (UBPV-6) genome (accession number: NC_030402, [18]. The remaining contigs were 73–79% identical to both bovine parvovirus-1 and UBPV-6. The taxonomy of this viral family has changed, with bovine parvovirus-1 being placed within the *Ungulate bocaparvovirus 1* species grouping and UBPV-6 being designated as a separate species within the *Bocaparvovirus* genus [46].

- *Erythroparvovirus*

A 343 nt contig corresponding to the putative capsid protein of bovine parvovirus 3 was identified as having 97% nucleotide sequence identity to the reference bovine parvovirus-3 isolate (accession number: MG026727). This virus belongs to the *Ungulate erythroparvovirus 1* species [46];

- *Copiparvovirus*

There were 22 contigs which were assembled to produce a consensus sequence covering approximately 91% of the genome and with 99.4% nucleotide identity to Bosavirus MS-2016a (Accession number NC_031959; total length 5371 nt) [47].

#### 3.1.7. Polyomaviridae

Five contigs were identified with sequence identity to bovine polyomavirus. The polyomavirus sequences in the current study had 99.6% identity to bovine polyomavirus 2a (Accession number KX455486). This virus has the documented types 1, 2, 2a, 2b, and 3. For comparison the sequence from the current study was found to have 83.1% nucleotide identity with type 2b (Accession number KM496325).

#### 3.1.8. Papillomaviridae

A 234 nt sequence was identified with 100% sequence identity to bovine papillomavirus 10 (Accession number KF017607). The region sequenced correlated with the E1 protein. This papillomavirus clustered within the genus *Xipapillomavirus*.

### 3.2. Case–Control Study of Virus Detection: BRD Cases versus Control Animals

Before the case–control study was conducted, some preliminary information on the frequency of detection of each of the RNA viruses identified in the NGS dataset was obtained from 60 nasal swab samples from cattle with BRD. These swabs were collected as part of the NBRDI [7]. Of these samples, 23 (38%) were positive for BNV, 17 (28%) were positive for BRVA, one (1.7%) sample was positive for BRBV, and three (5%) samples were positive for IDV. The newly designed primers and probe demonstrated no cross reactivity with other pathogens such as BoHV-1, BVDV-1, BPI3, BCoV, and BRSV.

In order to further evaluate the potential associations between the viruses detected in the NGS analysis and BRD, the extracts from the nasal swabs collected from the cattle with clinical signs of BRD (*n* = 141) and from matched, healthy cattle (*n* = 147) were tested for the presence of the genomic material of 10 viruses using qPCR assays [34]. A summary of the positive and negative qPCR results for each virus of interest is shown in Table 5. A complete list of the qPCR results for the cases and controls is provided in Supplemental Tables S3 and S4, respectively.

In total, the viruses were detected 113 times in the samples from 96 animals. When all animals were considered (cases and controls), BNV was the most frequent virus detected, accounting for 25.6% of the viruses detected, followed by IDV (23%) and BoHV-1 (16.8%). In the cases, a positive virus result was obtained 73 times in the samples from 61 animals. For these cases, BoHV-1 was the most frequently detected virus, representing 26% of all viruses detected, followed by IDV (22%) and BNV (20.5%). Among the controls, a virus was detected 40 times in 35 animals. BNV was the predominant viral pathogen detected (35%), followed by IDV (25%) and BRAV (22.5%) in the control group.

At least one virus was detected in 33.3% (96) of the animals, with 43.3% (61/141) for the BRD cases and 23.8% (35/147) for the controls. In order to evaluate if there were any associations between those animals testing positive for the viruses of interest and BRD, the odds ratios and their 95% confidence intervals were estimated (Table 5). The presence of at least one virus was significantly associated with clinical signs of BRD (*p* = 0.0005). Analyses of how each virus affected the risk of an animal being diagnosed with BRD showed that BoHV-1 was the only virus significantly associated with this disease. The animals testing positive for BoHV-1 were 47 (2.8–785.8, 95%CI, *p* = 0.007) times more likely to be diagnosed with BRD (Table 5). The presence of more than one virus was observed in 16 animals (5.5%), representing 7.8% of the cases (*n* = 11) and 3.4% of the controls (*n* = 5) (Supplemental Tables S3 and S4).

In order to compare the relative amounts of virus detected between the cases and controls, the Ct values (indicative of the amount of virus in a sample) between the two groups were compared. No significant differences were identified between the mean Ct values from samples collected from the cattle with clinical signs of BRD in comparison to the asymptomatic cattle for any of the viruses (Supplemental Table S5). For all viruses, except BoHV-1, BVDV-1, and BPI-3 (for which no virus was detected in the healthy control animals), an overlap was observed between the ranges of the Ct values between the BRD cases and the control animals. Additionally, when IDV was detected, there were no significant differences in the mean IDV Ct values for the animals with coinfections or those infected with IDV alone for the BRD cases, the controls, or all animals (Supplemental Table S6).

## 4. Discussion

Bovine respiratory disease is a major health problem for the beef cattle industry around the world, causing severe economic losses [1,48,49]. The disease has a complex aetiology, with the interaction between multiple pathogens, hosts, management, and environmental factors all contributing to the risk of disease. The availability of NGS-based viral metagenomics in recent years has provided a powerful tool for the large-scale and unbiased detection of known viruses and the discovery of unknown viruses in BRD-affected animals [14,18,50]. This study was no exception, with viruses from a number of different families detected in the nasal swabs of feedlot cattle affected by BRD. Several of these viruses have not been detected previously in Australian cattle, such as IDV, BRAV, BRBV, BNV, and UBPV-6. The detection of these viruses agrees with other virome studies of cattle with BRD [14,18,50,51].

At least one virus was detected in 33.3% of cattle (43.3% of cases and 23.8% of the controls), and the presence of one or more viruses in an individual was shown to be significantly associated with BRD, supporting the important role viruses play in the pathogenesis of this complex disease. The three most common viruses detected in the cases were BoHV-1, IDV, and BNV. Two of these viruses, IDV and BNV, in addition to BRAV, were the most frequently detected viruses in the control animals.

In the case–control study, BoHV-1 was the only virus found to be significantly associated with BRD, with BoHV-1-positive animals being 47 times more likely to be diagnosed with the disease (Table 5). The OR estimate was very imprecise, suggesting that the association between BoHV-1 and BRD risk was highly confounded. An important confounder of this result is that cattle were vaccinated with a modified live intranasal BoHV-1 vaccine on entry to the feedlot [34,52]. There is insufficient data to determine whether the detected BoHV-1 is the vaccine or a field strain of the virus. When considering that all the animals were vaccinated at the same time, and the case and control animals were matched, there were no BoHV-1-positive animals among the controls, suggesting the animals had cleared the vaccine at the time of BRD diagnosis. Other viruses were unlikely to have contributed to clinical signs in the BoHV-1-positive animals, as only three of the 19 BoHV-1-positive animals were coinfected with another virus (all with Ct values greater than 33) (Supplemental Tables S3 and S4). The exclusion of these BoHV-1-positive animals from the dataset marginally reduced the BRD risk. Similar to the current study, Hay et al. [3] also reported that cattle vaccinated at feedlot entry with the same BoHV-1 vaccine were at increased risk of developing BRD, OR = 6.0 (0.6–24.4 95% Credible Interval) [3]. Meanwhile, Hay et al. [3], with a large study population (*n* > 35,000), suggested that the OR estimate was confounded by the highly clustered application of the vaccine within the study population at the feedlot level (*n* = 14). As feedlots in the study either did or did not use the live vaccine, there was insufficient statistical power to further investigate this effect. The authors suggested that randomised controlled trials were required to examine this effect. The current study provides further weight to the need for such trials, as do other studies. Zhang et al. [50] reported that animals testing positive for BRSV had a greatly increased risk of BRD (OR = 13.422, 1.454–123.885, 95%CI, *p* = 0.022) compared to healthy animals. These animals were vaccinated at induction, with modified live vaccines, for BoHV-1, BVDV, BRSV, and BPI3. Similar to the study of Hay et al. [6], the imprecise risk estimate suggests high levels of confounding which requires further investigation to elucidate the underpinning mechanisms.

BNV was the most frequently detected virus in this study (10.1% of all animals; 25.6% of all viruses). This virus was first reported in cattle with BRD in a US feedlot in 2013 [28]. In the current study, a full-length genome was assembled, and although this was only 85.9% identical to the reference genome, the phylogenetic analysis revealed that it clustered with the reference isolate in the *Tobaniviridae* family (subfamily *Remotovirinae,* genus *Bostavirus*). As BNV is a recently emerging virus, its clinical significance is yet to be clearly defined. In the initial report, there was no conclusive data to associate the virus with illness as it was not the only viral agent identified, and healthy cattle were not available for comparison [28]. Interestingly, BNV-positive cattle were found to be 12.8 times (OR = 0.078, 95%CI 0.021–0.288, *p* = 0.000) less likely to be diagnosed with BRD in a Canadian feedlot [50]. A similar trend was observed in Mexican feedlots, albeit in fewer animals, where 3.7% and 11.5% of BRD cases and controls tested positive for BNV, respectively [18]. In the current study, despite being the most prevalent virus detected, no association with BRD was detected, with the positive samples being evenly distributed among the case (10.6%) and control animals (9.5%). Coinfections with BNV were also detected in this study, with 6 out of 15 for the cases and 2 out of 14 for the controls. No difference in the average Ct values between the cases or controls infected with BNV was observed, suggesting the lack of an association with the disease was not due to virus titre at the time of sampling (Supplemental Table S5).

It is evident that several aspects of BNV and its association with BRD warrant further investigation. Given the limited amount of sequence data available for this virus, further sequencing of positive samples is required to accurately characterise its genome sequence and organisation. Additionally, more data on the relationship of this virus with the clinical signs of BRD is required, particularly due to the observed association with a reduced risk of the disease reported in a previous study [50] and the comparatively high number of positive samples from control animals in the current study.

Parvoviruses are recognised as important pathogens in various groups of mammals; however, there are few published studies with respect to the clinical significance of these viruses in cattle. Two members of the family *Parvoviridae* from the genus *Bocaparvovirus,* bovine parvovirus 1 and UPBV-6, were detected in the current study. Bovine parvoviruses have been reported to be associated with respiratory and gastrointestinal diseases in cattle [53]. Despite this, the role of UBPV-6 in the pathogenesis of BRD remains unclear as it has been detected with high frequency in healthy cattle [18,50]. However, in the present study, the BRD cases tended to be 4.4 times (*p* = 0.07) more likely to have the virus than the control animals. Moreover, Zhang et al. [50] reported that cattle positive for UBPV-6 were 3.4 times (OR = 0.296, 95%CI 0.108–0.814, *p* = 0.019) less likely to be diagnosed with BRD. In the current study, UBPV-6 was detected in more of the cases (5.6%) than the controls (1.4%), although this positive association with BRD was not statistically significant. Additional viruses from the *Parvoviridae* family were also detected in this study. BPV3 and bosavirus are usually observed as a contaminant of commercial bovine serum, although BPV3 has also been detected in cattle in Brazil; however, there was no evidence to support its association with clinical disease [54,55].

It would seem implausible that either BNV or UBPV-6 provide specific protection from BRD, rather their association with reduced risk of disease may represent an unperturbed state of the respiratory microbiota, where, in a healthy animal, the presence of some viruses is, if not commensal, benign. This hypothesis is consistent with the changing paradigm that mucosal surfaces are not sterile, suggesting that research should be equally focused on characterising the microbiomes of healthy animals as well as diseased animals to better understand the pathogenesis of complex diseases such as BRD.

IDV is the most recently discovered member of the *Orthomyxoviridae* family and is the first influenza virus to be associated with cattle, the species considered to be the natural reservoir of this virus [56]. IDV sequences were identified in both the cases (11.3%) and the controls (6.8%) in this study. A total of 6 out of the 16 cases with IDV infection were coinfected with one or two other viruses (BRSV once, UBPV-6 twice, BNV twice, BRSV, and BNV once), whilst, in the controls, two of the five animals were coinfected (BRAV and BNV). This virus is being increasingly detected around the world, although there are conflicting reports with respect to its association with BRD. IDV is found predominantly in the upper respiratory tract of cattle and is generally associated with mild to moderate respiratory disease [18,57]. It has also been reported in asymptomatic animals, which was observed in the current study [18,51]. This could be attributed to the fact that cattle, being the natural reservoirs of this virus, may be more likely to carry the virus without displaying clinical signs of disease.

It has been proposed that IDV may contribute to BRD by exacerbating the effects of coinfecting pathogens because of the changes it induces in the upper respiratory tract [14,57,58,59]. IDV has been more commonly detected in cattle coinfected with other pathogens [59,60], and higher IDV loads have also been reported in symptomatic cattle in which multiple viruses were detected in comparison to those infected with IDV alone [57,58]. In the current study, there were no associations between IDV viral loads or coinfections and Ct values, suggesting no significant differences between those animals coinfected or solely infected with IDV (in the cases, controls, and all animals. Additionally, no difference in Ct values was observed between the IDV detected in the cases and the IDV detected in the controls.

Rhinitis viruses (BRAV and BRBV) were also detected in this study. These viruses have not been reported before in Australian cattle, although they are being reported more frequently in published studies [14,18,29,50,51]. Rhinitis viruses have also been found to have an inconsistent association with BRD, and it has been suggested that other factors may be required for the disease to develop in cattle infected with these viruses [14,18,50]. At least two serotypes for rhinitis A have been reported, which may be a contributing factor to the reported differences in pathogenicity. In the current study, although BRAV was detected more frequently in the controls than in the cases (6.1% vs. 2.1%), this was not a statistically significant association. Further research is required to determine if there are any associations between these viruses and BRD in feedlot cattle.

There were other viruses detected in this study that were considered unlikely to play important roles in BRD development. Similarly to bosavirus and BPV3, bovine polyomavirus is usually considered to be a contaminant in tissue culture serum. However, recent studies have implicated bovine polyomavirus 1 and 2 in kidney and nonsuppurative encephalitis in cattle, respectively [61,62]. Bovine papilloma virus 10 has been associated with cutaneous papillomas in cattle [63].

As with previous reports, the current study has identified a wide repertoire of viruses in both BRD-affected and -unaffected cattle. Collectively, the BRD virome studies highlight the power of applying NGS as an unbiased diagnostic tool to detect the presence/absence of known and unknown viruses. These studies also highlight that the detection of a virus or viruses does not equate to causality with respect to the disease of interest. Viruses, particularly BNV, BRAV, and IDV, were detected in 23.8% of asymptomatic cattle in the current study. To date, these three viruses have been reported to have variable associations with BRD [14,18,50,51], and therefore associations with the disease, when detected in symptomatic cattle remain problematic when the current paradigm suggests that viruses are pathogens that cause disease. The detection of a virus in asymptomatic animals could also be due to subclinical infections, the detection of the virus in the disease incubation period before the onset of clinical signs, or the continued shedding of the virus once the clinical signs have resolved. Asymptomatic carriers potentially pose a significant risk to a herd with respect to transmission to susceptible animals. It is also difficult to draw conclusions with respect to these emerging viruses, as information on the role they play in the pathogenesis of BRD and other diseases, if any, is yet to be defined.

With the increasing use of NGS technologies, known, emerging, and novel viruses are being detected and identified more frequently in healthy people and animals. Therefore, consideration should also be given to a potential commensal or at least the nonclinical role(s) of these viruses in the respiratory system [64]. Of particular note are the associations of BNV and UBPV-6 with BRD, which warrant further investigation due to their significant association with reduced risk of disease reported in previous studies and the higher number of positive samples for these viruses in the control animals from the current study [50]. Clearly, more data on the relationship of these viruses in animals without BRD are required. It would seem improbable that BNV or other viruses provide specific protection from BRD, rather their association with a reduced risk of disease may represent an unperturbed state of the respiratory virome, where, in a healthy animal, the presence of these and perhaps other viruses are, if not commensal, benign. The interaction of viruses with commensal microbiota (particularly bacteria but also fungi), in addition to the composition of the commensal microbiota at the time of infection, may influence disease outcomes in individuals. The commensal microbiota is known to influence the health of the host. Preliminary research has examined the role viruses have on the commensal microbiota, with both positive and negative outcomes documented, including both the exacerbation and suppression of viral infections [65]. This work has been predominantly conducted in humans, with a focus on the gut microbiome, although there is some evidence to support similar interactions between bacteria and viruses within the human respiratory tract, too [64]. These findings would be expected to occur in animals too, but research is required to evaluate these interactions. This suggests that research should be equally focused on characterising the viromes and microbiomes in healthy animals to better understand the pathogenesis of complex diseases such as BRD.

Hick et al. [66] reported the detection and isolation of BCoV in Australian cattle affected by BRD, and BCoV has also been associated with BRD mortality in Australian feedlot cattle [67]. The current study reports the first complete genome sequence for BCoV from the Australian cattle population. Previous studies have reported that the specific genotypes of other BRD-associated viruses, BoHV-1, BVDV-1, and BPI3, circulate within this population, perhaps a consequence of this country’s strict quarantine controls [45,68,69,70]. The phylogenetic analyses completed in the current study demonstrated the robust clustering of the ORF1ab gene sequence with homologous BCoV sequences from other countries with no evidence for a specific linage detected (Figure 2). Unlike many other viruses with RNA genomes (e.g., BVDV-1), the coronavirus genome replication complex has a proofreading capacity that is likely to contribute to stable genome replication over time without external selection factors, such as vaccination [71].

One potential limitation of the current study is the use of nasal swabs to sample the mucosa of the nasopharyngeal when compared to longer swabs to sample the oropharyngeal mucosa, or even the sampling of the lower respiratory tract via bronchial lavage. Nasal swabs were used in the current study as they represent a robust and less invasive method of collecting respiratory samples under field conditions. Zhang et al. [50] compared the detection of several viruses in nasal swabs and tracheal washes collected from BRD-affected cattle, concluding that there was poor correlation between the two sample types. As an example, BRAV and bovine adenovirus 3 were only detected in the nasal swabs; the remaining viruses were detected in both sample types, albeit at different frequencies. None of the viruses of interest were detected in the tracheal washes only [50]. McDaneld et al. [72] evaluated the bacterial populations sampled with nasal swabs and deep nasopharyngeal swabs from BRD-affected and heathy cattle, concluding that both types of swabs yielded similar results. These previous studies, together with the results of the current study, suggest that nasal swabs are a very good sample for the detection of BRD-associated pathogens in affected cattle.

The pooled samples were utilised to generate the sequencing libraries to identify the RNA viruses of interest in the first phase of this study. The possibility that viruses at very low concentrations were not detected in the sequencing cannot be excluded due to the dilution effect of pooling. However, the pooling of samples has proven to be a robust method to efficiently increase the number of samples that can be evaluated for identifying pathogens [73,74]. Nagy et al. [75] were able to expand the repertoire of viruses associated with BRD in affected Egyptian cattle using a pool of 43 nasal swabs. In comparison, the pool of six samples utilised in the current study is considered to be a balanced and conservative approach. Furthermore, the consistency of the range of viruses detected in the pools of the current study with those published in similar studies suggests the minimal loss, if any, of information on the viruses present in individual samples from the sample pool size utilised in this study.

## 5. Conclusions

In conclusion, the results of the present study are similar to those reported by other BRD studies, confirming the complexity of the virome in cattle with and without BRD, highlighting the need for further research to clearly define the roles, if any, of a suite of emerging viruses in the pathogenesis of BRD. Future research should also aim to elucidate the importance of the presence of viruses in healthy animals, as this may provide insights into dysbiosis, which leads to disease. Improved knowledge of the viruses involved with BRD in cattle will inform the implementation of management and preventative strategies, including informing the development of diagnostic tests and vaccines aimed at reducing the impact of this economically important disease within the intensive finishing sectors of the global beef industry.

## Figures and Tables

**Table 1:** Oligonucleotide pairs and dual-labelled hydrolysis probe sequences used for quantitative real-time PCR detection of the viruses.

Target Pathogen	Name	Primer/Probe Sequence 5′-3′ ^1^
Bovine nidovirus	BNV_Fwd	GTCAACTGGAGTAGGTCGAAAG
	BNV_Rev	TCAGCCTCATTCCTAACATCAC
	BNV_Probe	TEX615-AGGTACCATTACTATACTGAGCTGGCAGC-BHQ-2
Bovine rhinitis A virus	BRAV_Fwd	AGGTACCCGGAGGTAACAA
	BRAV_Rev	GGTGCCTGATGAGACATAGAAG
	BRAV_Probe	6FAM-CCCAGGTCAGATCCAGAGTGTCAC-BHQ-1
Bovine rhinitis B virus	BRBV_Fwd	GCGATTGTGTCCTAGGGTTT
	BRBV_Rev	GCCACTGAGGTTAGCTTCTC
	BRBV_Probe	Cy5-CTGTCCTTTGCACGGCGTGG-BHQ-2 ^2^
Influenza D virus	IDV_Fwd	GAGGAATGCTGATGGGAATGT
	IDV_Reverse	CTTTGTAGCCCAGTCCAGTAAC
	IDV_Probe	HEX-ATTACAGGGAGGAAGCATTGGCCA-BHQ-1
Ungulate bocaparvovirus 6	UBPV6_Fwd	GGGAAGAGTGGCTTCAGTTTAG
	UBPV6_Rev	GGCTCTTCTCCTTGTTCTTCTG
	UBPV6_Probe	HEX-TCCAGATACAATCAGAAGAAGCGCCA-ZEN/IABkFQ

^1^ Fluorophores and quenchers are shown at the 5′ and 3′ termini, respectively, of each probe sequence. ^2^ Primers and probe sequences from [32].

**Table 2:** Summary of the bovine influenza D virus partial genomic segment sequences identified in the current study, as compared to the strain IDV D/bovine/Miyazaki/B22/2016.

		IDV Sequence Data (Current Study)					IDV Reference	
Segment	Protein	Length (nt)	Coverage (%)	Nucleotide Identity (%)	Amino Acid Similarity (%)	GenBank	Length (nt)	Reference Accession
1	polymerase PB2	1054	44.6%	98.0	99.2	OQ348274	2364	LC270265.1
2	polymerase PB1	929	39.9%	98.5	99.7	OQ348275	2330	LC270266.1
3	polymerase 3 P3	918	41.0%	98.4	98.7	OQ348276	2195	LC270267.1
4	haemagglutinin-esterase HE	248	12.1%	94.8	92.7	OQ348277	2049	LC270268.1
5	nucleoprotein	NT ^1^					1775	LC270269.1
6	P42	NT ^1^					1219	LC270270.1
7	non-structural protein 2	NT ^1^					868	LC270271.1

^1^ Not detected.

**Table 3:** Comparison of the Australian bovine coronavirus (BCoV-Aus) genome reconstructed in this study with next-generation sequencing data to the BCoV reference strain BCoV-ENT and the buffalo coronavirus (BuCoV) strain B1-28F.

Open Reading Frame	BCoV-Aus Bases	BCoV-ENT Bases; Identity (%)	BuCoV B1-28F Bases; Identity (%)
Complete genome	30,999	31,028; 98.9	30,985; 98.5
orf 1ab polyprotein	21,278	21,284; 99.1	21,284; 98.4
32 kDa nonstructural protein	837	837; 98.7	837; 98.1
haemaglutinin esterase (HE)	1275	1275; 99.1	1275; 97.7
spike structural protein (S)	4092	4092; 98.3	4092; 98.8
4.9 kDa nonstructural protein	89	90; 92.2	78; 100
4.8 kDa nonstructural protein	142	138; 85.6	135; 90.2
12.7 kDa nonstructural protein	330	330; 98.5	330; 99.4
small membrane protein (E)	255	255; 100	255; 99.6
matrix protein (M)	693	693; 98.7	693; 100
nucleocapsid protein (N)	1347	1347; 98.7	1347; 99.5
internal protein (I)	624	624; 98.7	624; 99.5

**Table 4:** Comparison of the Australian bovine nidovirus (BNV-Aus) genome sequence to the reference BNV genome sequence of strain TCH5.

Characteristic	BNV-Aus	BNV TCH5	Nucleotide Identity (%)	Amino Acid Similarity (%)
complete genome	20,262	20,261	85.9	
replicase polyprotein (pp1a/b)	15,323	15,332	87.2	90.5
glycoprotein S (S)	1686	1689	81.9	83.5
membrane protein 1 (M1)	696	696	87.2	91.8
nucleocapsid (N)	534	537	85.8	86.5
glycoprotein G2 (G2)	1371	1368	75.6	64.1
hypothetical protein	267	267	94.8	92.0

**Table 5:** Summary of the viral risk factors in the BRD case–control study. The qPCR-based detection of 10 viruses in the nasal swabs from cattle diagnosed with BRD (cases) and healthy cattle (controls) are summarised. Estimated odds ratios (OR) for the effect a positive qPCR result on the risk of the cattle being diagnosed with BRD. The 95% confidence intervals (95%CI) are shown for the risk factors, along with *p* values, with values <0.05 indicating a significant association.

Risk Factor	qPCR Result	Cases (%)	Controls (%)	OR	95%CI	*p* Value
Infected with one or more virus	Positive	61 (43.3)	35 (23.8)	2.4	1.5–4.0	0.0005
	Negative	80 (56.7)	112 (76.2)			
Bovine herpesvirus 1	Positive	19 (13.5)	0 (0)	47	2.8–785.8	0.0074
	Negative	122 (86.5)	147 (100)			
Bovine coronavirus	Positive	2 (1.4)	3 (2.0)	0.7	0.1–4.2	0.7
	Negative	139 (98.6)	144 (98.0)			
Bovine respiratory syncytial virus	Positive	6 (4.3)	2 (1.4)	3.2	0.6–16.2	0.2
	Negative	135 (95.7)	147 (98.6)			
Bovine parainfluenza virus	Positive	1 (0.7)	0 (0)	3.1	0.1–78	0.5
	Negative	140 (99.3)	147 (100)			
Bovine viral diarrhoea virus 1	Positive	3 (2.1)	0 (0)	7.5	0.4–145.6	0.2
	Negative	138 (97.9)	147 (100)			
Influenza D virus	Positive	16 (11.3)	10 (6.8)	1.8	0.8–4.0	0.2
	Negative	125 (88.7)	137 (93.2)			
Bovine rhinitis A virus	Positive	3 (2.1)	9 (6.1)	0.3	0.1–1.3	0.1
	Negative	138 (97.9)	138 (93.9)			
Bovine rhinitis B virus	Positive	0 (0)	0 (0)	Not carried out	-	-
	Negative	141 (100)	147(100)			
Bovine nidovirus	Positive	15 (10.6)	14 (9.5)	1.1	0.5–2.4	0.8
	Negative	126 (89.4)	133 (90.5)			
Ungulate bocaparvovirus 6	Positive	8 (5.7)	2 (1.4)	4.4	0.9–20.9	0.07
	Negative	133 (94.3)	145 (98.6)			

## Data Availability

The raw sequencing data generated in this study are available in the NCBI Sequence Read Archive database (BioProject number PRJNA926021. The completed genome sequences for bovine coronavirus (BoCV-Aus) and bovine nidovirus (BNV-Aus) completed in this study, were deposited in the NCBI GenBank database and assigned the accession numbers OQ348268 and OQ348282, respectively. Partial viral genome sequences for other the viruses described in this study were deposited in the NCBI GenBank database and assigned the accession numbers in parenthesis: bovine influenza D virus ASRA (Segment 1, OQ348274; Segment 2; OQ348275, Segment 3; OQ348276, Segment 4; OQ348277), bovine viral diarrhoea virus 1 (OQ348280, OQ348281 ), bovine rhinitis A virus (OQ348269), bovine rhinitis B virus (OQ348270), ungulate bocaparvovirus 1 (OQ348279), bovine parvovirus 3 (OQ348271), bosavirus (OQ348278), bovine papillomavirus 10 (OQ348271) and bovine polyomavirus 2a (OQ348273).

## Supplementary Materials

The following supporting information can be downloaded at: https://www.mdpi.com/article/10.3390/v15020455/s1, Figure S1. Schematic representation of the annotated bovine coronavirus genome sequence determined in this study. Predicted coding sequences (CDS) are shown and named using the nomenclature of the polypeptides they encode. Figure S2. Schematic representation of the annotated bovine nidovirus genome determined in this study. Predicted coding sequences (CDS) are shown and named using the nomenclature of the polypeptides they encode. Table S1. Oligonucleotide pairs used for PCR amplification and direct amplicon sequencing to resolve gaps and/or regions of low sequence coverage in the bovine coronavirus (BCoV) genome following the assembly of next generation sequencing data. Table S2. Oligonucleotide pairs used for PCR amplification and direct amplicon sequencing to resolve gaps and/or regions of low sequence coverage in the bovine Nidovirus (BNV) genome following the assembly of next-generation sequencing data. Table S3. Quantitative real-time PCR threshold cycle (Ct) values for cattle diagnosed with bovine respiratory disease (BRD cases, BC) for the case/control study. The CT values are shown for the respective viruses where the value was ≤35 was deemed positive. Blank cells indicate a negative result. Table S4. Quantitative real-time PCR threshold cycle (Ct) values for cattle not diagnosed with bovine respiratory disease (controls, C) for the case/control study. The Ct values are shown for the respective viruses where the value was ≤35 was deemed positive. Blank cells indicate a negative result. Table S5. Comparison of the threshold cycle (Ct) values from the quantitative real-time PCR analyses of extracts from nasal swab from feedlot cattle diagnosed with bovine respiratory disease (case) and health cattle (control). The results for bovine coronavirus (BCoV), bovine respiratory syncytial virus (BRSV), influenza D virus (IDV), bovine rhinitis A virus (BRAV), bovine nidovirus (BNV), and ungulate bocaparvovirus 6 (UBPV6) are shown. Table S6. Comparison of the threshold cycle (Ct) values from the quantitative real-time PCR analysis for influenza D virus (IDV) of extracts from nasal swab from feedlot cattle diagnosed with bovine respiratory disease (case) and health cattle (control). Comparison of the extract Ct values in cattle with viral co-infections and those with IDV alone are shown.
