# Supplementary material for: Characterisation of the Upper Respiratory Tract Virome of Feedlot Cattle and Its Association with Bovine Respiratory Disease
Source: Viruses. 2023 Feb 6;15(2):455. doi: 10.3390/v15020455 (PMC9961997; doi:10.3390/v15020455)
Supplement: Supplementary file 1 [file viruses-15-00455-s001.zip › Supplemental Files.pdf]

Supplementary Figures

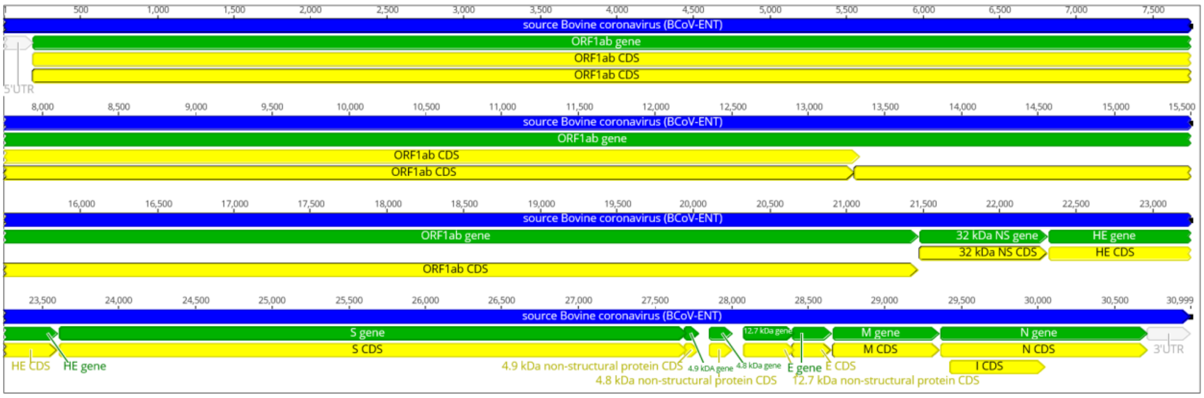

Supplemental Figure S1. Schematic representation of the annotated bovine coronavirus genome sequence determined in this study. Predicted coding sequences (CDS) are shown and named using the nomenclature of the polypeptides they encode are illustrated.

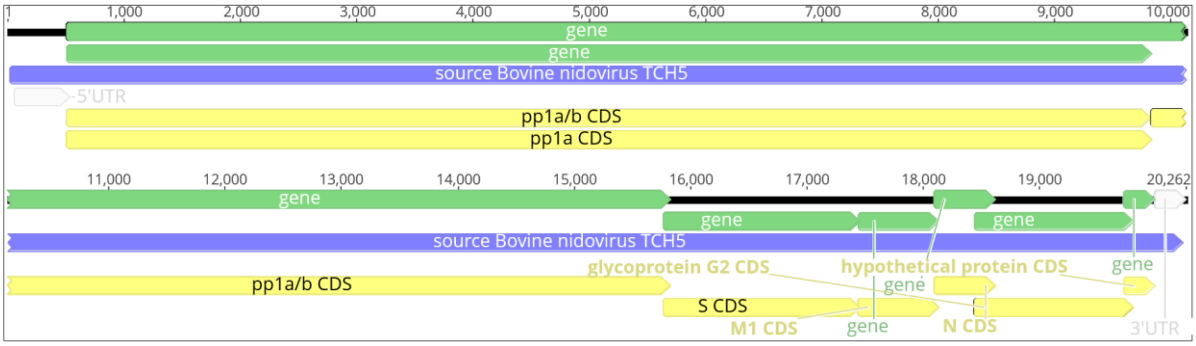

Supplemental Figure S2. Schematic representation of the annotated bovine nidovirus genome determined in this study. Predicted coding sequences (CDS) are shown and named using the nomenclature of the polypeptides they encode are illustrated.

Supplementary Tables

Supplemental Table S1. Oligonucleotide pairs used for PCR amplification and direct amplicon sequencing to resolve gaps and/or regions of low sequence coverage in the bovine coronavirus (BCoV) genome following the assembly of next generation sequencing data.

Pair ID	Name	Primer sequence (5′ to 3′)	Amplicon size (bases)
**BCoV 1**	Forward	GCATCCCGCTTCACTGAT	235
	Reverse	ATTCTGGAGCCCAGTGTAGTT	
**BCoV 2**	Forward	ATGAGGCTGGCGAGGAA	206
	Reverse	CATCATCCAAAGTGTCCTCTAAGAT	
**BCoV 3**	Forward	GAGCAAGCTTCCCTCAATTCT	1408
	Reverse	GAGCGGCCTCAACAGTAATAA	
**BCoV 4**	Forward	CTACTGCTAATACTGGTACGTCTG	1294
	Reverse	GCATATACCCTCATCAGCTTCC	
**BCoV 5**	Forward	TCCTAACACGGTTGATTGTCAG	193
	Reverse	CCACATCCATATT C/A ATCACAATACC	
**BCoV 6**	Forward	CCCTCTGGTAATTATTTAGCCATTTC	2596
	Reverse	AACACGACCACTATAGCAACTAC	
**BCoV 7**	Forward	ACTAGTAAACCTGCAATGCC	260
	Reverse	CTCAGGTTTCCAGATGTCCATTA	
**BCoV 8**	Forward	CCACTAAGCCACAGCAAGTA	844
	Reverse	TGGTAACTTAACATGCTGGCT	

Supplemental Table S2: Oligonucleotide pairs used for PCR amplification and direct amplicon sequencing to resolve gaps and/or regions of low sequence coverage in the bovine Nidovirus (BNV) genome following the assembly of next generation sequencing data.

Pair ID	Name	Sequence (5′ to 3′)	Amplicon size (bases)
**BNV 1**	Forward	CACCAATAGATTAGTCAAGCTGT	1638
	Reverse	ACCTGAAACGCACAGCCATA	
**BNV 2**	Forward	TATGGYTGYGCGTTTCAGGT	2428
	Reverse	GCTTTCGCTGTCCTCCTTCT	
**BNV 3**	Forward	CGCAGAAGTCGGTGTAAGAA	1607
	Reverse	CACCATTGATGCTTACTGCAC	
**BNV 4**	Forward	GGATGGTGCAGTAAGCATCA	2145
	Reverse	ACATGCCCACTCGGTGTAAG	
**BNV 5**	Forward	CTTACACCGAGTGGGCATGT	1032
	Reverse	TGACTTCAACGACCGACCCA	
**Bnido6**	Forward	TGAGGGTGAGACTTGCGAAC	2086
	Reverse	GTAACTGGTGCTCCRCAATT	
**BNV7**	Forward	AAGTTTGCCGCAGATGGAGT	2017
	Reverse	CCGCAAATCTCACAAGCTGC	
**BNV 8**	Forward	ACAGGTGGTACAAGTAGCGG	1513
	Reverse	CATGGGTGGGTGCCACATAA	
**BNV 9**	Forward	AGATTGGTCACRACCTGTGC	2518
	Reverse	CTGGCAAYTCAGGARGGCTAA	
**BNV 10**	Forward	TATGGGTTTAGCCATCCTGA	1776
	Reverse	GTCAACTGATACATACTATCACC	
**BNV 11**	Forward	GGTGATAGTATGTATCAGTTGAC	1417
	Reverse	TACCGCGCCCATTAGACATC	
**BNV 12**	Forward	GATGTCTAATGGGCGCGGTA	1889
	Reverse	TACTTCTTCGCACYAGGCGA	

Supplemental Table S3. Quantitative real-time PCR threshold cycle (Ct) values for cattle diagnosed with bovine respiratory disease (BRD cases, BC) for the case/control study. The CT values are shown for the respective viruses where the value was ≤35 was deemed positive. Blank cells indicate a negative result.

Animal number	status	BoHV-1	BCoV	BRSV	BPI-3	BVDV-1	IDV	BRAV	BRBV	BNV	UBPV-6
BC1	case		31.95								
BC2	case										
BC3	case										
BC4	case				28.09						
BC5	case	26.49									
BC6	case	32.18									
BC7	case	33.38									
BC8	case										
BC9	case	27.66									
BC10	case										
BC11	case	27.55									
BC12	case	25.34									
BC13	case						27.92				
BC14	case										
BC15	case						27.46				
BC16	case						31.33				
BC17	case										
BC18	case										
BC19	case						29.74				
BC20	case						29.87				25.87
BC21	case										
BC22	case										
BC23	case										

BC24	case				33.53			
BC25	case							33.74
BC26	case							
BC27	case							
BC28	case				25.5			
BC29	case							
BC30	case							
BC31	case				30.98			
BC32	case							
BC33	case				34.11			
BC34	case							30.9
BC35	case		28.64					
BC36	case							
BC37	case							
BC38	case		29.86					
BC39	case							
BC40	case							
BC41	case							
BC42	case	32.41		30.14				
BC43	case							
BC44	case						30.18	
BC45	case		33.32		23.76			
BC46	case							29.73
BC47	case							
BC48	case							36.73
BC49	case							
BC50	case							
BC51	case					34.02	33.99	
BC52	case							33.18
BC53	case							

BC54	case				32.98		
BC55	case						
BC56	case				30.5		
BC57	case						
BC58	case	32.84					33.12
BC59	case						
BC60	case						38.86
BC61	case						
BC62	case						
BC63	case						
BC64	case			30.69			
BC65	case					32.76	
BC66	case						
BC67	case						34.2
BC68	case						
BC69	case						
BC70	case						
BC71	case						
BC72	case						
BC73	case						
BC74	case		29.45				
BC75	case			28.1			25.01
BC76	case						
BC77	case						
BC78	case						
BC79	case	30.31					
BC80	case						
BC81	case						
BC82	case						
BC83	case						

BC84	case					
BC85	case					
BC86	case					
BC87	case					
BC88	case					
BC89	case					
BC90	case					
BC91	case					33.89
BC92	case					
BC93	case					31.97
BC94	case					
BC95	case					34.93
BC96	case					
BC97	case		34.35		21.41	30.58
BC98	case	33.58				
BC99	case					
BC100	case		34.63			
BC101	case				32.64	24.83
BC102	case	29.6				
BC103	case					
BC104	case					
BC105	case	32.41				
BC106	case					
BC107	case			33.1		
BC108	case					
BC109	case	29.7				34.58
BC110	case	33.72				
BC111	case					
BC112	case					33.94
BC113	case				31.38	28.86

BC114	case				
BC115	case				
BC116	case				
BC117	case				
BC118	case				
BC119	case		31.3		
BC120	case	34.45			
BC121	case	31.24			34.29
BC122	case				
BC123	case				
BC124	case	28.69			
BC125	case	29.33			
BC126	case				
BC127	case	29.66			
BC128	case				
BC129	case				
BC130	case			32.05	
BC131	case				
BC132	case				33.81
BC133	case				32.8
BC134	case				32.68
BC135	case				
BC136	case				
BC137	case	28.65			
BC138	case				
BC139	case				
BC140	case				
BC141	case				

Supplemental Table S4. Quantitative real-time PCR threshold cycle (CT) values for cattle not diagnosed with bovine respiratory disease (controls, C) for the case/control study. The CT values are shown for the respective viruses where the value was ≤35 was deemed positive. Blank cells indicate a negative result.

Animal number	Disease status	BoHV-1	BCoV	BRSV	BPI-3	BVDV-1	IDV	BRAV	BRBV	BNV	UBPV-6
C1	control										
C2	control		31.47								
C3	control										
C4	control										
C5	control										
C6	control										
C7	control										
C8	control										
C9	control										
C10	control										
C11	control										
C12	control										
C13	control						23.4				
C14	control										
C15	control										
C16	control										
C17	control										
C18	control										
C19	control										
C20	control										
C21	control						25.32				
C22	control						34.72				
C23	control						22.38				
C24	control						33.81				
C25	control										

C26	control									
C27	control									
C28	control									
C29	control	33.28								
C30	control									
C31	control									
C32	control									
C33	control		34.04							28.89
C34	control					31.41				
C35	control									
C36	control						31.35			
C37	control									
C38	control						29.01			
C39	control									
C40	control									
C41	control									
C42	control									
C43	control					30.75				
C44	control		34.46							28.49
C45	control									
C46	control					21.71				
C47	control									
C48	control									
C49	control									
C50	control									
C51	control									
C52	control									
C53	control									
C54	control									
C55	control									

C56	control
C57	control
C58	control
C59	control
C60	control
C61	control
C62	control
C63	control
C64	control
C65	control
C66	control
C67	control
C68	control
C69	control
C70	control
C71	control
C72	control
C73	control
C74	control
C75	control
C76	control
C77	control
C78	control
C79	control
C80	control
C81	control
C82	control
C83	control
C84	control
C85	control

C86	control			
C87	control			
C88	control			
C89	control			34.1
C90	control			
C91	control			
C92	control			
C93	control	35.1	27.22	
C94	control	25.28	32.23	
C95	control			
C96	control			
C97	control		34.09	
C98	control			
C99	control			
C100	control			34.7
C101	control			
C102	control			
C103	control			
C104	control			30.24
C105	control			
C106	control			
C107	control			29.61
C108	control		27.03	28.63
C109	control			
C110	control			
C111	control			
C112	control			
C113	control			
C114	control			
C115	control			

C116	control								34.33
C117	control								
C118	control								
C119	control								
C120	control								33.91
C121	control								34.85
C122	control								
C123	control								33.47
C124	control								
C125	control								
C126	control	31.16							
C127	control								
C128	control								
C129	control								
C130	control					28.94			34.38
C131	control								
C132	control								
C133	control								
C134	control						31.75		
C135	control								
C136	control								
C137	control						33		
C138	control								29.41
C139	control								30.83
C140	control								
C141	control								
C142	control								
C143	control								
C144	control								34.01
C145	control								34.36

C146	control	32.73
C147	control	

Supplemental Table S5. Comparison of the threshold cycle (Ct) values from the quantitative real-time PCR analyses of extracts from nasal swab from feedlot cattle diagnosed with bovine respiratory disease (case) and health cattle (control). The results for bovine coronavirus (BCoV), bovine respiratory syncytial virus (BRSV), influenza D virus (IDV), bovine rhinitis A virus (BRAV), bovine nidovirus (BNV), and ungulate bocaparvovirus 6 (UBPV6) are shown.

	**Viruses**											
	**BCoV**		**BRSV**		**IDV**		**BRAV**		**BNV**		**UBPV6**	
	case	*control*	*case*	*control*	*case*	*control*	*case*	*control*	*case*	*control*	*case*	*control*
Mean	32.18	31.97	31.70833	34.25	29.40438	27.772	32.5	30.93444	32.27267	32.63071	30.71875	28.69
Variance	0.1058	1.3111	7.208617	0.0882	12.49379	22.81757	3.2704	6.588703	7.30145	5.308376	12.88307	0.08
Observations	2	3	6	2	16	10	3	9	15	14	8	2
Hypothesised Mean Difference	0		0		0		0		0		0	
df	2		5		15		5		27		7	
t Stat	0.30002		-2.27741		0.932768		1.159763		-0.38477		1.579196	
P(T<=t) one-tail	0.396236		0.035885		0.182856		0.149256		0.351712		0.07915	
t Critical one-tail	2.919986		2.015048		1.75305		2.015048		1.703288		1.894579	
P(T<=t) two-tail	0.792472		0.071771		0.365712		0.298512		0.703424		0.158301	
t Critical two-tail	4.302653		2.570582		2.13145		2.570582		2.051831		2.364624	

Supplemental Table S6. Comparison of the threshold cycle (Ct) values from the quantitative real-time PCR analysis for influenza D virus (IDV) of extracts from nasal swab from feedlot cattle diagnosed with bovine respiratory disease (case) and health cattle (control). Comparison of the extract Ct values in cattle with viral co-infections and those with IDV alone are shown.

	**IDV total**		**IDV cases**		**IDV controls**	
	co-infection	*IDV only*	*co-infection*	*IDV only*	*co-infection*	*IDV only*
Mean	27.6725	29.33157895	27.86	30.34545455	27.11	27.9375
Variance	15.04150714	19.28873626	19.5498	12.27774727	6.6978	28.22353571
Observations	8	19	6	11	2	8
Pooled Variance	18.09951211		14.70176485		25.53281875	
Hypothesized Mean Difference	0		0		0	
df	25		15		8	
t Stat	-0.925280757		-1.277228486		- 0.207146983	
P(T<=t) one-tail	0.181832999		0.110469077		0.420535289	
t Critical one-tail	1.708140761		1.753050356		1.859548038	
P(T<=t) two-tail	0.363665998		0.220938153		0.841070577	
t Critical two-tail	2.059538553		2.131449546		2.306004135	
